# Radiomics-based nomogram as predictive model for prognosis of hepatocellular carcinoma with portal vein tumor thrombosis receiving radiotherapy

**DOI:** 10.3389/fonc.2022.906498

**Published:** 2022-09-20

**Authors:** Yu-Ming Huang, Tsang-En Wang, Ming-Jen Chen, Ching-Chung Lin, Ching-Wei Chang, Hung-Chi Tai, Shih-Ming Hsu, Yu-Jen Chen

**Affiliations:** ^1^ Department of Radiation Oncology, Taipei Hospital, Ministry of Health and Welfare, New Taipei City, Taiwan; ^2^ Department of Medicine, MacKay Medical College, New Taipei City, Taiwan; ^3^ Department of Biomedical Imaging and Radiological Sciences, National Yang Ming Chiao Tung University, Taipei, Taiwan; ^4^ Division of Gastroenterology, Department of Internal Medicine, MacKay Memorial Hospital, Taipei, Taiwan; ^5^ Department of Artificial Intelligence and Medical Application, MacKay Junior College of Medicine, Nursing, and Management, New Taipei City, Taiwan; ^6^ Department of Radiation Oncology, MacKay Memorial Hospital, Taipei, Taiwan; ^7^ Department of Medical Research, MacKay Memorial Hospital, Taipei, Taiwan; ^8^ Department of Medical Research, China Medical University Hospital, Taichung, Taiwan

**Keywords:** hepatocellular carcinoma, portal vein tumor thrombosis, radiation therapy, radiomics, predictive model

## Abstract

**Background:**

This study aims to establish and validate a predictive model based on radiomics features, clinical features, and radiation therapy (RT) dosimetric parameters for overall survival (OS) in hepatocellular carcinoma (HCC) patients treated with RT for portal vein tumor thrombosis (PVTT).

**Methods:**

We retrospectively reviewed 131 patients. Patients were randomly divided into the training (*n* = 105) and validation (*n* = 26) cohorts. The clinical target volume was contoured on pre-RT computed tomography images and 48 textural features were extracted. The least absolute shrinkage and selection operator regression was used to determine the radiomics score (rad-score). A nomogram based on rad-score, clinical features, and dosimetric parameters was developed using the results of multivariate regression analysis. The predictive nomogram was evaluated using Harrell’s concordance index (C-index), area under the curve (AUC), and calibration curve.

**Results:**

Two radiomics features were extracted to calculate the rad-score for the prediction of OS. The radiomics-based nomogram had better performance than the clinical nomogram for the prediction of OS, with a C-index of 0.73 (95% CI, 0.67–0.79) and an AUC of 0.71 (95% CI, 0.62–0.79). The predictive accuracy was assessed by a calibration curve.

**Conclusion:**

The radiomics-based predictive model significantly improved OS prediction in HCC patients treated with RT for PVTT.

## Introduction

Hepatocellular carcinoma (HCC) is the sixth most common cancer and the third leading cause of cancer death worldwide. The prognosis of HCC is poor, with a 5-year survival rate of 5%–18% (1–4). Approximately 70% of newly diagnosed HCC patients are not suitable for curative local treatment (5). The major cause is macrovascular invasion, in which tumor cells invade the portal vein, hepatic vein, or the inferior vena cava in the liver (6). Portal vein tumor thrombosis (PVTT) is a common complication of HCC and is related to poor prognosis and poor response to local treatment. The incidence of PVTT in HCC ranges from 44% to 62% (7). PVTT can interfere with the portal blood supply in the normal liver and deteriorate liver function. It may contribute to intrahepatic or extrahepatic metastasis (8). This locally advanced and mostly unresectable disease is associated with rapid cancer progression and deterioration of liver function. Patients with PVTT have a median survival rate of only 3 months without treatment (9). Current treatments for HCC with PVTT include targeted therapy with sorafenib and lenvatinib and locoregional treatments such as operation (OP), radiation therapy (RT), transarterial chemoembolization (TACE), and transarterial radioembolization (TARE) (10–13). However, there is no consensus on the best forms of treatment for HCC patients with PVTT. Several clinical studies have reported that RT alone or combined with TACE is an effective treatment for HCC with PVTT (14–17). The clinical target volume (CTV) of RT for PVTT usually encompasses the area of PVTT and/or visible tumor with a 5–10–mm margin to cover the involved portal vein region (18). The advantages of RT for HCC with PVTT are local tumor control, portal vein patency, and survival benefit (19). No universal marker or method of clinical utility that can predict the survival of HCC patients treated with RT for PVTT is known. An effective predictive model that may guide precision medicine for these patients with generally poor survival is required.

HCC can be diagnosed on contrast-enhanced computed tomography (CT) or magnetic resonance imaging (MRI) (20). Therefore, HCC is frequently diagnosed on images alone, precluding the requirement for tissue proof. Currently, CT is routinely used by physicians for diagnosis, staging, and RT planning for HCC. Radiomics is an emerging and promising methodology for medical image analysis that converts medical images into high-dimensional quantitative features using machine learning algorithms and statistical analysis software. Thus, it may facilitate the detection of lesions (21, 22), improve diagnostic accuracy (23–25), predict disease risk and prognosis (26–32), evaluate the risk of treatment and treatment-related toxicities (33–37), and guide treatment strategies (38, 39) in different types of diseases, especially malignancies. Several studies have been published on the use of radiomics in HCC (40–43). Wang et al. analyzed the prognostic value of MRI textural features in HCC in 201 patients who underwent OP (44). Meng et al. integrated intratumoral and peritumoral CT radiomics features and clinical features to develop and validate a radiomics-based predictive nomogram to predict overall survival (OS) in HCC patients undergoing TACE (45). Cozzi et al. appraised the ability of a radiomics-based analysis to predict local response and OS in HCC patients who were eligible for curative or palliative RT (46). To the best of our knowledge, relatively limited data and few studies focused on prognosis estimation in HCC patients treated with RT for PVTT with radiomics analysis are available. This study uses radiomics features of CTV, which are derived from the pre-RT CT of HCC patients with PVTT, in combination with clinical features and RT dosimetric parameters to develop a predictive model for HCC with PVTT.

## Material and methods

### Patients

We retrospectively reviewed HCC patients newly diagnosed with PVTT between December 2007 and December 2019 in one institution. A contrast-enhanced CT or MRI was performed for diagnosis and staging. According to the 7th edition American Joint Committee on Cancer/American Joint Committee on Cancer staging system, all patients were staged IIIB (patients with a single tumor or multiple tumors of any size involving a major branch of the portal vein or hepatic vein, Vp4 in Liver Cancer Study Group of Japan classification). All patients had an Eastern Cooperative Oncology Group (ECOG) performance status of 0 to 2. In this study, patients were either inoperable or not eligible for TACE or TARE. The primary treatments were RT and/or targeted therapy. Patients with a history of OP, RT, TACE, or TARE were excluded. A total of 131 patients were enrolled and randomly divided into the training cohort (*n* = 105) and validation cohort (*n* = 26), with a ratio of 4:1.

### RT protocol

Patients underwent CT simulation in the supine position and were immobilized with an alpha cradle. Planning CT images with a slice thickness of 3 mm were acquired through the entire upper abdomen. Contrast-enhanced CT was used to localize the PVTT along with the primary tumor and to assess the enhancement patterns of lesions. The gross tumor volume (GTV) was delineated using the diagnostic and simulation images of the PVTT with or without the primary liver tumor. The CTV was determined by expanding the GTV margin by 5–10 mm to consider areas at significant risk of microscopic disease. The planning target volume (PTV) was generated by adding a 5–10–mm margin to the CTV in all directions for a setup error. RT was delivered using either three-dimensional conformal radiotherapy or intensity-modulated radiation therapy (IMRT) based on physician preference. The treatment plans were designed using 6- or 10-MV photons. All patients were treated with linear accelerators. Dosimetric parameters such as the dose of the CTV and normal organs were extracted from RT planning systems (Eclipse Treatment Planning System; Varian Medical Systems Inc., Palo Alto, CA, USA). The prescribed dose was 45, 50, or 60 Gy delivered in 1.8–2 Gy per fraction (BED10: 53.1–72.0 Gy). The goals were to deliver the prescribed dose to ≥95% of the PTV and 95% of the prescribed dose to ≥99% of the PTV. The dosimetric parameters were recorded for evaluation. After RT, abdominal CT or MRI was performed for response assessment. Most patients underwent abdominal CT or MRI 1 month after RT. The patency status of the portal vein area was evaluated by experienced radiologists.

### Acquisition of CT images

Contrast-enhanced CT was performed using Philips MRC 800 (Philips Medical Systems, Amsterdam, Netherlands) with a peak tube voltage of 120 kVp, tube current of 325 mA, rotation time of 0.75 s, matrix of 512 × 512, field of view of 50 cm, and slice thickness of 3 mm for RT planning and radiomics analysis.

### Texture analysis

**Figure 1:**
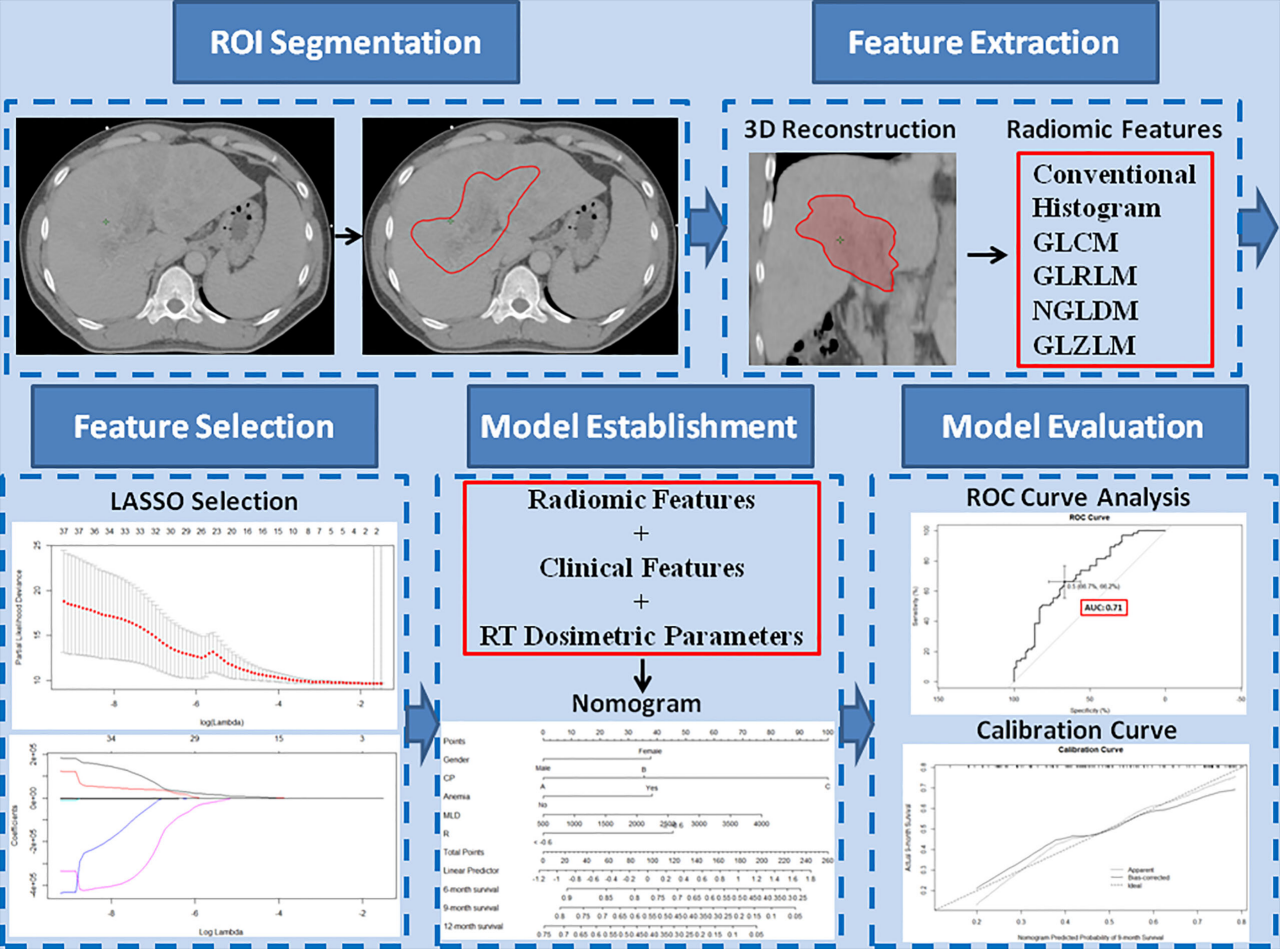
Study workflow. The region of interest (ROI) was segmented on all transverse contrast-enhanced computed tomography images by experienced radiation oncologists using the Eclipse system. After a three-dimensional reconstruction of the ROI, 48 textural features, including conventional features, histogram features, gray-level co-occurrence matrix, gray-level run-length matrix, neighborhood gray-level dependence matrix, and gray-level zone length matrix, were extracted. The extracted features were selected by least absolute shrinkage and selection operator regression. Based on the selected radiomics features, clinical features, and radiation therapy dosimetric parameters, a nomogram model was established to predict overall survival. The performance of the predictive model was evaluated with concordance index, area under the curve of the receiver operating characteristic curve, and calibration curve.

The CTV, the region of interest (ROI), was contoured by experienced radiation oncologists on all axial CT images. Segmentation was performed using the Eclipse system. Three-dimension ROI was visualized using Local Image Features Extraction (LIFEx) version 5.10 (http://www.lifexsoft.org; Orsay, France) (47). The LIFEx software was used to extract the textural features of the ROI. A total of 48 textural features of the images were extracted, including features of a histogram-based matrix, gray-level co-occurrence matrix (GLCM), gray-level run length matrix (GLRLM), neighborhood gray-level dependence matrix (NGLDM), and gray-level zone length matrix (GLZLM) (**Figure 1**).

### Extraction of radiomics features

The study population was divided into the training and validation cohorts in a ratio of 4:1 using the sample function of R (version 3.6.1) software (https://www.r-project.org; Vienna, Austria) to make randomization. The least absolute shrinkage and selection operator (LASSO) Cox regression was performed to determine the radiomics features that can predict OS in the training cohort. We performed the 10-fold cross-validation 20 times. The final value of lambda (penalized parameter) was determined with the minimized mean deviance and the corresponding subset of covariates with non-zero coefficients. Features were selected by the total times of non-zero coefficient in 20 randomized 10-fold cross-validations. The Cox proportional-hazard model was fitted with the selected features, and the radiomics score (rad-score) predicting OS could be calculated linearly.

### Clinical feature extraction

The following 17 clinical features were selected: age, gender, etiology of viral hepatitis, drinking history, ECOG performance status, Child-Pugh class, tumor size, anemia status, serum levels of alpha-fetoprotein (AFP), white blood cell, platelet, albumin, alanine aminotransferase, aspartate aminotransferase (AST), total bilirubin, creatinine, and prothrombin time.

### RT dosimetric parameters

**Figure 2:**
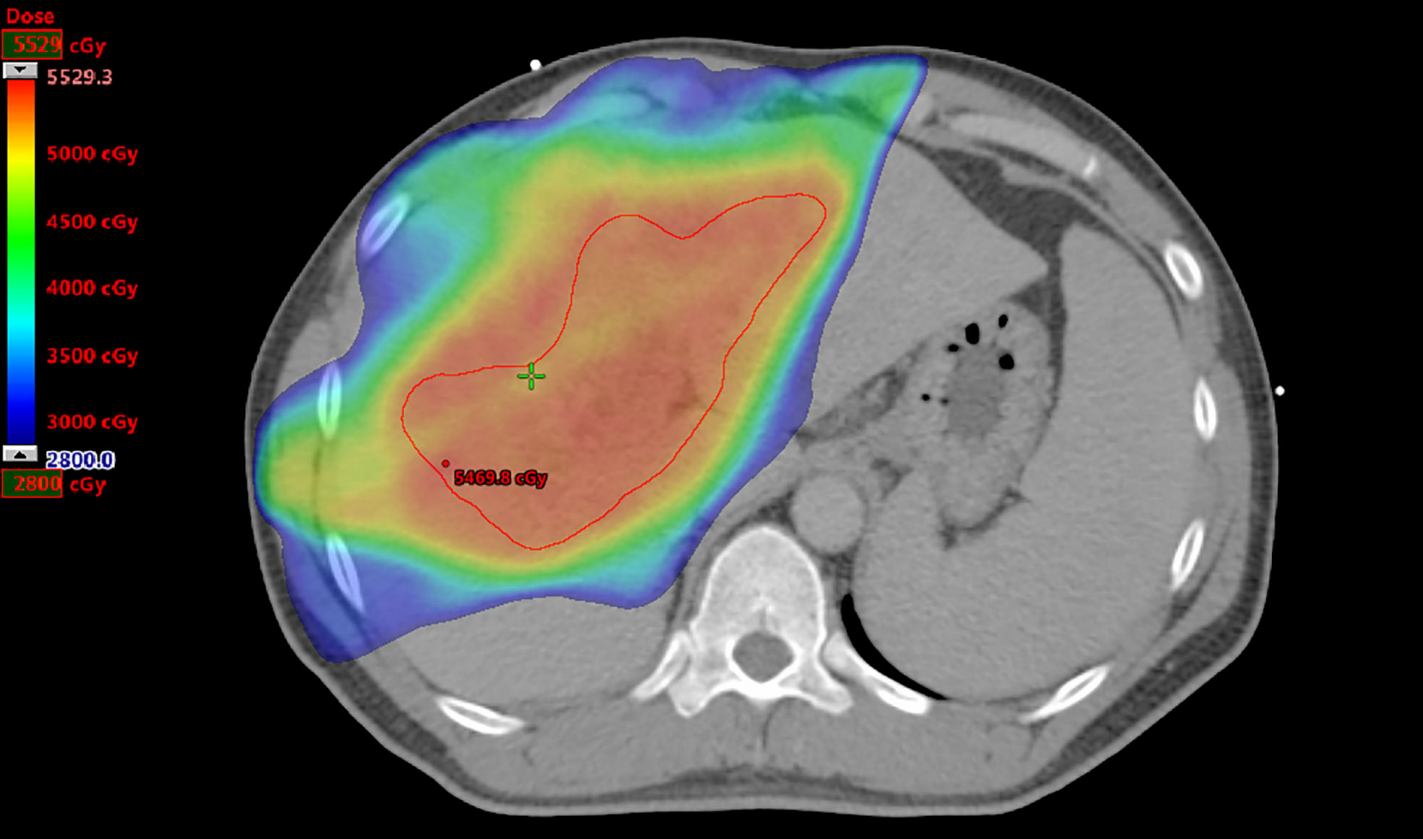
Radiation therapy plan for a patient. The prescribed dose to treat portal vein tumor thrombosis only is 50 Gy. The clinical target volume as the region of interest is contoured in red, and the volume is 280.7 cm^3^. The normal liver volume is 1,769.0 cm^3^, and the mean liver dose is 2,189.4 cGy.

The prescribed RT doses, RT fields as involved PVTT with or without primary liver tumors, CTV, normal liver volume (NLV), and mean liver doses (MLDs) of all patients were recorded (**Figure 2**).

### Statistical analysis

Statistical analysis was performed using R (version 3.6.1) and SPSS version 24.0 (IBM Corporation, Armonk, NY, USA). Numerical data are presented as mean ± standard deviation. LASSO regression analysis was performed using the “glmnet” package to select the radiomics features for rad-score to predict OS. The optimal cutoff value of the rad-score was determined using X-tile software (Yale University, New Haven, CT, USA). The survival curves were plotted using the Kaplan–Meier method and assessed using the log-rank test. The Chi-square test was used to assess categorical variables, and the Mann–Whitney *U* test was used to assess continuous variables. Univariate Cox regression analysis was performed to determine the predictors of OS from rad-score, clinical features, and RT dosimetric parameters. Thereafter, multivariate Cox regression analysis was used to select prognostic factors for the establishment of predictive nomogram models. The “survival” and “rms” packages were used for survival analysis, nomogram model construction, Harrell’s concordance index (C-index) calculation, and calibration curve. The C-index was a measure of goodness-of-fit for outcomes in a regression model, ranging from 0.5 to 1. A C-index value of 0.5 indicated that the predictive ability of the model was no better than a random chance, whereas C-index values of >0.7 and >0.8 indicated that the model was good and strong, respectively. A value of 1 implied that the model perfectly predicted the outcome. The “survivalROC” package was used for calculation and comparison of the area under the curve (AUC) of the receiver operating characteristic (ROC) curve for evaluation of the nomogram. The AUC ranged from 0.5 to 1. The discrimination potent of the model based on the value of AUC was as follows: 0.5, no discrimination potent; 0.7–0.8, acceptable; 0.8–0.9, excellent; and >0.9, outstanding. Differences were considered significant at *p* < 0.05.

### Establishment of predictive models

Based on the results of multivariate Cox regression analysis, the nomogram models with significant clinical features, RT dosimetric parameters, and/or rad-score were constructed to predict OS in HCC patients treated with RT for PVTT. The confirmation of nomograms was subjected to a 1,000 resampling bootstrap analysis for validation. The predictive models were evaluated with C-indexes, AUC of ROC curves, and calibration curves (**Figure 1**).

### Ethical statement

This study was approved by the Institutional Review Board of our institution (IRB number: 20MMHIS215e).

## Results

### Patient characteristics

A summary of the baseline characteristics of the 131 patients is presented in **Table 1**. The median age at diagnosis was 61 years (range: 36–87 years), and 108 (82.4%) of the patients were men. A total of 110 (84.0%) patients had hepatitis B/C virus infection, and 93 (71.0%) patients had a drinking history. In this study, 83, 44, and 4 patients had Child–Pugh classes A, B, and C, respectively. The pre-RT tumor size was 9.5 ± 5.2 cm. Before RT, 59 (45.0%) patients had anemia, and the median serum AFP level was 149.3 ng/ml (range: 1.2–515,800.0 ng/ml). The baseline characteristics of the training and validation cohorts are summarized in **Table 2**. No significant differences were found in the baseline characteristics of the two cohorts.

**Table 1:** Baseline characteristics of all patients.

Characteristics	*N* = 131
Age (median (range), year)	61 (36–87)
Gender (*N* (%))	
Male	108 (82.4)
Female	23 (17.6)
Hepatitis (B/C) (*N* (%))	
Yes	110 (84.0)
No	21 (16.0)
Drinking history (*N* (%))	
Yes	93 (71.0)
No	38 (29.0)
ECOG (*N* (%))	
0	36 (27.5)
1	71 (54.2)
2	24 (18.3)
Child–Pugh class (*N* (%))	
A	83 (63.4)
B	44 (33.6)
C	4 (3.0)
Tumor size (mean (SD), cm)	9.5 (5.2)
Anemia (*N* (%))	
Yes	59 (45.0)
No	72 (55.0)
AFP (median (range), ng/ml)	149.3 (1.2–515,800.0)
WBC (mean (SD), 10^3^/µl)	6.3 (2.5)
PLT (mean (SD), 10^3^/µl)	172.3 (105.5)
ALB (mean (SD), g/dl)	3.5 (0.6)
ALT (mean (SD), IU/L)	53.2 (42.1)
AST (mean (SD), IU/L)	86.0 (80.8)
TBIL (mean (SD), mg/dl)	1.5 (0.9)
Cr (mean (SD), mg/dl)	0.9 (0.3)
PT (mean (SD), s)	11.8 (1.0)

ECOG, Eastern Cooperative Oncology Group; SD, standard deviation; AFP, alpha-fetoprotein; WBC, white blood cell; PLT, platelet; ALB, albumin; ALT, alanine aminotransferase; AST, aspartate aminotransferase; TBIL, total bilirubin; Cr, creatinine; PT, prothrombin time.

The performance status was graded with the ECOG score, in which grade 0 indicated fully active, grade 1 indicated able to perform light work, and grade 2 indicated capable of all self-care but unable to perform any work activities.

**Table 2:** Baseline characteristics of the training and validation cohorts.

Characteristics	Training cohort (*N* = 105)	Validation cohort (*N* = 26)	*p*
Age (median (range), year)	61 (36–87)	62 (45–84)	0.36
Gender (*N* (%))			
Male	88 (83.8)	20 (76.9)	0.41
Female	17 (16.2)	6 (23.1)	
Etiology of viral hepatitis (*N* (%))			
Hepatitis B	64 (60.9)	12 (46.2)	0.11
Hepatitis C	23 (21.9)	4 (15.4)	
Hepatitis B + C	5 (4.8)	2 (7.7)	
None	13 (12.4)	8 (30.7)	
Drinking history (*N* (%))			
Yes	76 (72.4)	17 (65.4)	0.48
No	29 (27.6)	9 (34.6)	
ECOG (*N* (%))			
0	28 (26.7)	8 (30.7)	0.92
1	59 (56.2)	12 (46.2)	
2	18 (17.1)	6 (23.1)	
Child–Pugh class (*N* (%))			
A	68 (64.8)	15 (57.7)	0.79
B	34 (32.4)	10 (38.5)	
C	3 (2.8)	1 (3.8)	
Tumor size (mean (SD), cm)	9.5 (5.1)	9.4 (5.2)	0.93
Anemia (*N* (%))			
Yes	46 (43.8)	13 (50.0)	0.57
No	59 (56.2)	13 (50.0)	
AFP (median (range), ng/ml)	149.3 (1.2–515,800.0)	136.9 (2.0–121,480.0)	0.59
WBC (mean (SD), 10^3^/µl)	6.3 (2.4)	6.2 (2.7)	0.84
PLT (mean (SD), 10^3^/µl)	177.4 (108.7)	151.5 (89.9)	0.28
ALB (mean (SD), g/dl)	3.5 (0.6)	3.6 (0.6)	0.29
ALT (mean (SD), IU/L)	54.0 (43.9)	50.0 (33.8)	0.51
AST (mean (SD), IU/L)	87.1 (81.2)	81.5 (63.7)	0.64
TBIL (mean (SD), mg/dl)	1.5 (0.9)	1.5 (0.9)	0.85
Cr (mean (SD), mg/dl)	0.9 (0.3)	0.9 (0.2)	0.83
PT (mean (SD), s)	11.7 (1.0)	12.1 (1.2)	0.15

ECOG, Eastern Cooperative Oncology Group; SD, standard deviation; AFP, alpha-fetoprotein; WBC, white blood cell; PLT, platelet; ALB, albumin; ALT, alanine aminotransferase; AST, aspartate aminotransferase; TBIL, total bilirubin; Cr, creatinine; PT, prothrombin time.

The performance status was graded with the ECOG score. A two-sided p-value of < 0.05 was considered statistically significant.

### RT dosimetric parameters

The RT dosimetric parameters for 131 patients are presented in **Table 3**. A total of 25, 101, and 5 patients were treated with an RT dose of 45, 50, and 60 Gy, respectively. The RT field in 88 (67.2%) patients involved PVTT only and that in 43 (32.8%) patients involved PVTT and primary liver tumors. The median CTV was 164.6 cm^3^ (range: 19.5–2,189.0 cm^3^). The NLV was 1,140.7 ± 480.8 cm^3^, and the MLD was 1891.3 ± 651.5 cGy. The RT dosimetric parameters for the training and validation cohorts are summarized in **Table 4**. No significant differences were found between the two cohorts for RT dosimetric parameters.

**Table 3:** RT dosimetric parameters of all patients.

Parameters	*N* = 131
RT dose (*N* (%))	
45 Gy	25 (19.1)
50 Gy	101 (77.1)
60 Gy	5 (3.8)
RT field (*N* (%))	
Involved PVTT	88 (67.2)
Involved PVTT + liver tumors	43 (32.8)
CTV (median (range), cm^3^)	164.6 (19.5–2,189.0)
NLV (mean (SD), cm^3^)	1,140.7 (480.8)
MLD (mean (SD), cGy)	1,891.3 (651.5)

RT, radiation therapy; PVTT, portal vein tumor thrombosis; CTV, clinical target volume; NLV, normal liver volume; SD, standard deviation; MLD, mean liver dose.

**Table 4:** RT dosimetric parameters of the training and validation cohorts.

Parameters	Training cohort (*N* = 105)	Validation cohort (*N* = 26)	*p*
RT dose (*N* (%))			
45 Gy	21 (20.0)	4 (15.4)	0.87
50 Gy	80 (76.2)	21 (80.8)	
60 Gy	4 (3.8)	1 (3.8)	
RT field (*N* (%))			
Involved PVTT	72 (68.6)	16 (61.5)	0.49
PVTT + liver tumors	33 (31.4)	10 (38.5)	
CTV (median (range), cm^3^)	175.6 (27.3–2,189.0)	154.2 (19.5–1,958.0)	0.63
NLV (mean (SD), cm^3^)	1137.4 (498.2)	1,154.0 (411.2)	0.88
MLD (mean (SD), cGy)	1,895.5 (661.5)	1,874.5 (621.5)	0.88

RT, radiation therapy; PVTT, portal vein tumor thrombosis; CTV, clinical target volume; NLV, normal liver volume; SD, standard deviation; MLD, mean liver dose.

A two-sided p-value of < 0.05 was considered statistically significant.

### Treatment outcome

The treatment outcomes of the patients are presented in **Table 5**. The median follow-up time was 9.8 months (range, 1.6–57.9 months), and 101 (77.1%) patients underwent abdominal CT or MRI images for response assessment. Three (2.3%) patients were alive at the time of the current analysis. Sixteen (15.8%) patients had patent portal veins after RT. The median OS was 9.8 months (95% CI, 8.0–11.6 months), and the median progression-free survival (PFS) was 5.6 months (95% CI, 4.8–6.4 months). Distant metastases were found in 22 (16.8%) patients. The treatment outcomes of the training and validation cohorts are summarized in **Table 6**. No significant differences in treatment outcomes were found between the two cohorts.

**Table 5:** Treatment outcomes of all patients.

Outcomes	*N* = 131
Patency (*N* (%))	
Yes	16 (15.8)
No	85 (84.2)
OS (median (95% CI), m)	9.8 (8.0–11.6)
PFS (median (95% CI), m)	5.6 (4.8–6.4)
DM (*N* (%))	
Yes	22 (16.8)
No	109 (83.2)

OS, overall survival; CI, confidence interval; PFS, progression-free survival; DM, distant metastasis.

**Table 6:** Treatment outcomes of the training and validation cohorts.

Outcomes	Training cohort (*N* = 105)	Validation cohort (*N* = 26)	*p*
Patency (*N* (%))			
Yes	13 (15.5)	3 (17.6)	0.82
No	71 (84.5)	14 (82.4)	
OS (median (95% CI), m)	9.8 (7.9–11.7)	10.1 (6.7–13.7)	0.87
PFS (median (95% CI), m)	5.2 (4.4–6.1)	5.9 (4.1–7.7)	0.47
DM (*N* (%))			
Yes	19 (18.1)	3 (11.5)	0.64
No	86 (81.9)	23 (88.5)	

OS, overall survival; CI, confidence interval; PFS, progression-free survival; DM, distant metastasis.

A two-sided p-value of <0.05 was considered statistically significant.

### Radiomics feature extraction and development of the rad-score

**Figure 3:**
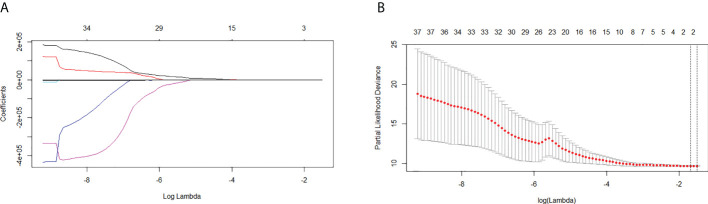
Least absolute shrinkage and selection operator (LASSO) regression analysis for the selection of significant radiomics features from the 48 textural features. **(A)** Coefficient profile of the LASSO model. **(B)** Optimal tuning parameter (lambda) selection using 10-fold cross-validation with minimum criteria. Two significant radiomics features were extracted.

A total of 48 radiomics features were extracted from the imaging data of all patients. Two features were selected by LASSO Cox regression analysis to predict the OS in the training cohort (**Figure 3**). The rad-score formula was GLRLM_HGRE × −2.973897e−05 + GLRLM_SRHGE × −2.504878e−05.

### Rad-score and correlation with OS

**Figure 4:**
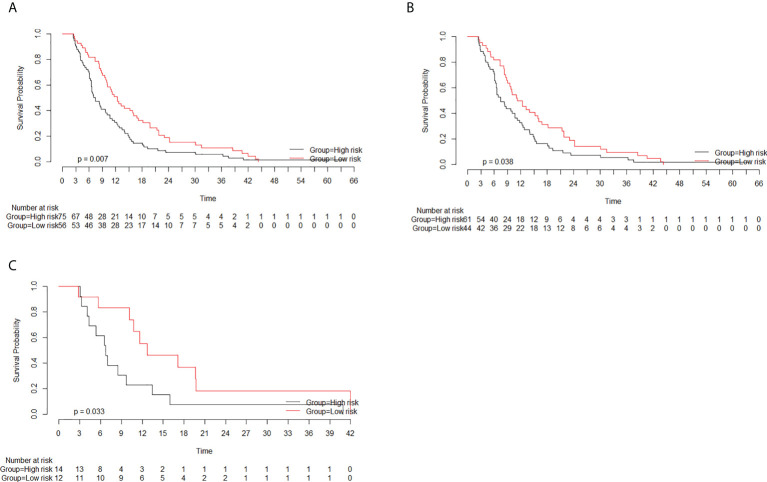
Survival curves of the high- and low-risk groups based on the radiomics score (rad-score) classification. The rad-scores in the high- and low-risk groups were more than −0.6 and less than −0.6, respectively. **(A)** Considering all patients, the median overall survival (OS) rates in the high- and low-risk groups were 7.4 months (95% CI, 6.5–10.7) and 12.4 months (95% CI, 10.0–16.8), respectively (*p* = 0.007). **(B)** Considering the training cohort, the median OS rates in the high- and low-risk groups were 7.5 months (95% CI, 6.5–11.2) and 11.8 months (95% CI, 9.6–16.8), respectively (*p* = 0.038). **(C)** Considering the validation cohort, the median OS rates in the high- and low-risk groups were 6.8 months (95% CI, 4.3–NA) and 12.6 months (95% CI, 10.7–NA), respectively (*p* = 0.033).

The optimal cutoff value of the rad-score, as determined by X-tile software, was −0.6. The patients were divided into the high- (≧−0.6) and low-risk (<−0.6) groups based on the cutoff value of the rad-score. The median OS rates in the high- and low-risk groups were 7.4 months (95% CI, 6.5–10.7) and 12.4 months (95% CI, 10.0–16.8), respectively (*p* = 0.007). Considering the training cohort, the median OS rates in the high- and low-risk groups were 7.5 months (95% CI, 6.5–11.2) and 11.8 months (95% CI, 9.6–16.8), respectively (*p* = 0.038). In the validation cohort, the median OS rates in the high- and low-risk groups were 6.8 months (95% CI, 4.3–NA) and 12.6 months (95% CI, 10.7–NA), respectively (*p* = 0.033). The median OS rates were significantly lower in the high-risk groups than in the low-risk groups in both the training and validation cohorts (**Figure 4**).

### Extraction of significant features

Univariate and multivariate Cox regression analyses were performed to determine the predictors of OS from rad-score, clinical features, and RT dosimetric parameters. Univariate analysis revealed seven predictors, namely gender, Child–Pugh class, anemia status, rad-score, MLD, tumor size, and AST, for OS prediction. Gender, Child–Pugh class, anemia status, rad-score, and MLD were found to be independent predictors in multivariate analysis (**Table 7**).

**Table 7:** Univariate and multivariate analyses for predictors of OS.

Predictors	Univariate analysis			Multivariate analysis		
	HR	95% CI	*p*	HR	95% CI	*p*
Gender						
Male	1			1		
Female	1.704	1.062–2.735	0.025	1.886	1.013–3.512	0.045
Child–Pugh class						
A	1			1		
B and C	1.515	1.042–2.201	0.028	1.672	1.053–2.655	0.029
Anemia						
No	1			1		
Yes	1.617	1.123–2.328	0.009	1.690	1.043–2.739	0.033
Rad-score						
<−0.6	1			1		
≧−0.6	1.635	1.137–2.351	0.008	1.540	1.007–2.355	0.047
MLD (cGy)	1.000	1.000–1.001	0.050	1.001	1.000–1.001	0.002
Tumor size (cm)	1.054	1.016–1.093	0.005	1.040	0.990–1.092	0.119
AST (U/L)	1.003	1.001–1.004	0.001	1.002	0.998–1.006	0.292

OS, overall survival; HR, hazard ratio; CI, confidence interval; rad-score, radiomics score; MLD, mean liver dose; AST, aspartate aminotransferase.

A two-sided p-value of <0.05 was considered statistically significant.

### Establishment of the predictive model

**Figure 5:**
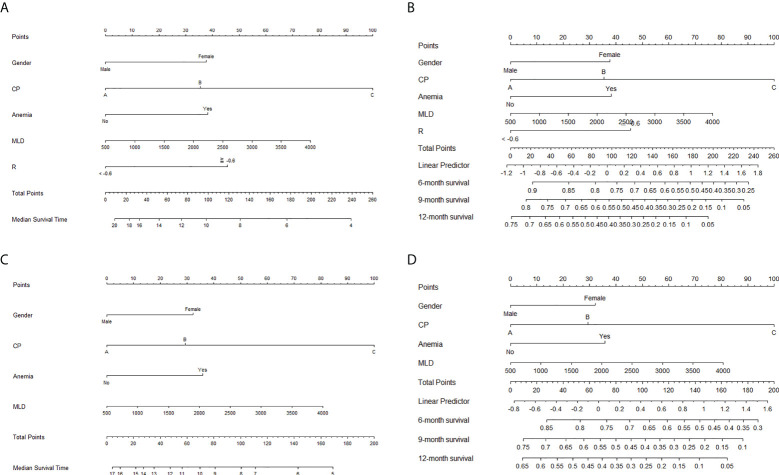
Nomograms for the prediction of overall survival. Nomograms with radiomics score, significant clinical features, and radiation therapy (RT) dosimetric parameters for the prediction of **(A)** median survival time and **(B)** 6-, 9-, and 12-month survival rates. Nomograms with selected clinical features and RT dosimetric parameters for the prediction of **(C)** median survival time and **(D)** 6-, 9-, and 12-month survival rates.

Based on the result of multivariate Cox regression analysis, a radiomics-based nomogram with significant clinical features, RT dosimetric parameters, and rad-score was developed to predict OS. A clinical nomogram with selected clinical features and RT dosimetric parameters was developed for OS prediction (**Figure 5**).

### Performances of different predictive nomograms and significant features

**Figure 6:**
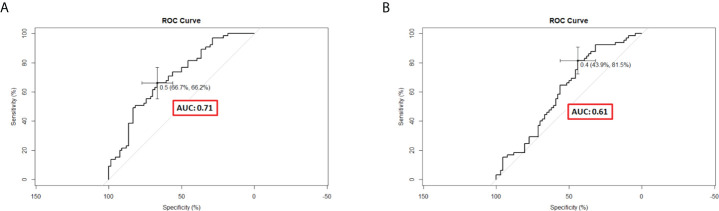
Receiver operating characteristic curves of different predictive nomograms for 9-month survival. **(A)** The area under the curve (AUC) was 0.71 (95% CI, 0.62–0.79) in the radiomics-based nomogram. **(B)** The AUC was 0.61 (95% CI, 0.51–0.71) in the clinical nomogram.

**Figure 7:**
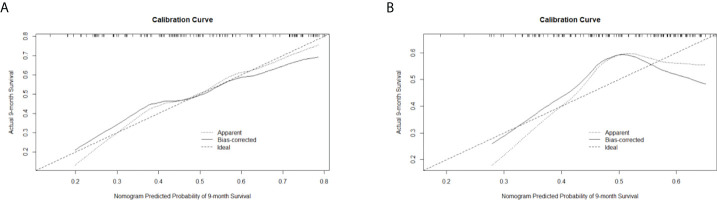
Calibration curves of **(A)** the radiomics-based nomogram and **(B)** clinical nomogram for the prediction of 9-month survival.

C-indexes were used to evaluate the discrimination power of significant features, clinical nomogram, and radiomics-based nomogram. The C-index profiles are presented in **Table 8**. In this study, the radiomics-based nomogram showed the best discrimination power, which was examined by internal validation. ROC analyses for 9-month survival, the AUCs for the radiomics-based nomogram and clinical nomogram were 0.71 (95% CI, 0.63–0.79) and 0.61 (95% CI, 0.51–0.71), respectively (**Figure 6**). The calibration curves of the radiomics-based nomogram and clinical nomogram are presented in **Figure 7**. The radiomics-based nomogram exhibited better predictive accuracy than the clinical nomogram for the prediction of 9-month survival.

**Table 8:** C-indexes of significant features, clinical nomogram, and radiomics-based nomogram.

Variables	Training cohort		Validation cohort		All patients	
	C-index	95% CI	C-index	95% CI	C-index	95% CI
Gender	0.54	0.50–0.58	0.53	0.41–0.65	0.54	0.50–0.58
Child–Pugh class	0.54	0.48–0.60	0.65	0.54–0.76	0.56	0.51–0.61
Anemia	0.56	0.50–0.62	0.58	0.46–0.70	0.56	0.51–0.61
MLD	0.51	0.44–0.58	0.59	0.46–0.72	0.53	0.47–0.59
Rad-score	0.57	0.52–0.62	0.64	0.53–0.75	0.58	0.53–0.63
Clinical nomogram	0.60	0.53–0.67	0.72	0.58–0.86	0.61	0.55–0.67
Radiomics-based nomogram	0.72	0.65–0.79	0.82	0.69–0.95	0.73	0.67–0.79

C-index, concordance index; CI, confidence interval; MLD, mean liver dose; rad-score, radiomics score.

## Discussion

In this study, we intended to develop a radiomics-based nomogram using pre-RT CT data. Univariate and multivariate analyses revealed that the rad-score significantly influenced OS. The performance of the radiomics-based nomogram was better than the clinical nomogram, and the predictive accuracy of each significant feature in the C-index and ROC analysis was examined by the calibration curve.

This study was conducted in the Department of Radiation Oncology in a medical center. All HCC patients were treated with the same RT protocol to ensure the standardization of the treatment and CT quality. CT was performed according to the American Association of Physicists in Medicine (AAPM) and American College of Radiology (ACR) guidelines (AAPM report #74 and #96 and ACR CT QC manual) and standard quality assurance measures.

Few studies have reported the application of radiomics and clinical features to predict treatment outcomes and prognosis in different types of cancers treated with RT. Hou et al. established an integrated model that combined posttreatment CT radiomics features and clinical features for response and OS prediction in esophageal cancer patients undergoing neoadjuvant chemoradiotherapy (48). Wu et al. developed a nomogram using radiomics and clinical features to predict OS in HCC patients treated with stereotactic body radiotherapy (SBRT) for PVTT (49). Parr et al. indicated that a radiomics-based predictive model combined with clinical features is better than an analysis of clinical features alone for predicting OS in pancreatic cancer patients treated with SBRT (50). Thus, radiomics combined with clinical features may have a better performance than analysis with clinical features alone for treatment response and OS prediction.

This study has several limitations. First, MRI is the preferred imaging modality for the evaluation of liver lesions. The technique of MRI-guided RT with MRI simulation and planning is rapidly developing (51). However, contrast-enhanced CT is still the main imaging methodology for diagnosis, staging, and RT planning for HCC, with acceptable sensitivity and high specificity. It is noninvasive, well-developed in current clinical practice, and not time-consuming or labor-consuming. Second, sorafenib has been used as the standard systemic treatment of advanced HCC during the investigation period of our study population. Currently, different agents such as lenvatinib, checkpoint inhibitors, and antivascular endothelial growth factor receptor antibodies have demonstrated efficacy in the treatment of advanced HCC. The effects of different systemic treatments should be examined in the future. Third, this study included 30 patients without CT or MRI follow-up data. Fourth, the small number of patients from a single institute could not draw a firm conclusion for application in other hospitals. In this retrospective review study, the standardization of CT simulation protocol, RT dose to PTV, and follow-up schedule lasted for 12 years, which might provide an informative database for analyzing radiomics and clinical outcomes. The current results may provide proof-of-concept information and practical procedures for other hospitals trying to apply radiomics in each institute. A prospective large-scale and multicenter study is required. Finally, the data in this study are derived from one hospital. Although internal validation was conducted for verification, further multicenter analysis is required for external validation.

## Conclusion

Radiomics features combined with clinical features and dosimetric parameters have better performance than each significant feature and clinical nomogram. This study recommends the development of a predictive model with significant clinical features, radiomics features, and dosimetric parameters. The multicenter analysis is warranted after the standardization of treatment protocol, radiology imaging, and radiomics data in all hospitals for external validation to ensure the accuracy of the universal predictive model.

## Data availability statement

The original contributions presented in the study are included in the article/supplementary material. Further inquiries can be directed to the corresponding authors.

## Ethics statement

The studies involving human participants were reviewed and approved by MacKay Memorial Hospital. The patients/participants provided their written informed consent to participate in this study.

## Author contributions

Conceptualization: Y-MH and Y-JC. Methodology: Y-MH, H-CT, S-MH, and Y-JC. Software: Y-MH Validation: Y-MH, S-MH, and Y-JC. Formal analysis: Y-MH. Investigation: Y-MH. Resources: Y-MH, T-EW, M-JC, C-CL, C-WC, and Y-JC. Data curation: Y-MH. Writing—original draft preparation: Y-MH. Writing—review and editing: Y-MH, S-MH, and Y-JC. Visualization: Y-MH. Supervision: Y-JC. Project administration: Y-JC. Funding acquisition: Y-JC. All authors have read and agreed to the published version of the manuscript.

## Funding

This research was funded by MacKay Memorial Hospital (Grant numbers MMH-E-109-13 and MMH-E-110-13) and the Ministry of Science and Technology of Taiwan (Grant number MOST 109-2314-B-195-003-MY3).

## Acknowledgments

We would like to thank Editage for providing their editing services.

## Conflict of interest

The authors declare that the research was conducted in the absence of any commercial or financial relationships that could be construed as a potential conflict of interest.

## Publisher’s note

All claims expressed in this article are solely those of the authors and do not necessarily represent those of their affiliated organizations, or those of the publisher, the editors and the reviewers. Any product that may be evaluated in this article, or claim that may be made by its manufacturer, is not guaranteed or endorsed by the publisher.
